# Clonal architecture and evolutionary history of Waldenström's macroglobulinemia at the single-cell level

**DOI:** 10.1242/dmm.050227

**Published:** 2023-08-23

**Authors:** Ramón García-Sanz, María García-Álvarez, Alejandro Medina, Elham Askari, Verónica González-Calle, María Casanova, Igor de la Torre-Loizaga, Fernando Escalante-Barrigón, Miguel Bastos-Boente, Abelardo Bárez, Nerea Vidaña-Bedera, José María Alonso, María Eugenia Sarasquete, Marcos González, María Carmen Chillón, Miguel Alcoceba, Cristina Jiménez

**Affiliations:** ^1^ Hematology Department, University Hospital of Salamanca (HUS/IBSAL), CIBERONC and Cancer Research Institute of Salamanca-IBMCC (USAL-CSIC), Salamanca 37007, Spain; ^2^ Hematology Department, Fundación Jiménez Díaz, Centro de Investigación Biomédica en Red-Cáncer, Madrid 28040, Spain; ^3^ Hematology Department, Hospital Costa del Sol, Marbella 29603, Spain; ^4^ Hematology Department, Complejo Asistencial Universitario de León, León 24071, Spain; ^5^ Hematology Department, Complejo Asistencial de Ávila, Ávila 05071, Spain; ^6^ Hematology Department, Complejo Asistencial Universitario de Palencia, Palencia 34005, Spain

**Keywords:** Disease mechanisms, Genomic alterations, Protein expression, Single-cell, Waldenström's macroglobulinemia

## Abstract

To provide insight into the subclonal architecture and co-dependency patterns of the alterations in Waldenström's macroglobulinemia (WM), we performed single-cell mutational and protein profiling of eight patients. A custom panel was designed to screen for mutations and copy number alterations at the single-cell level in samples taken from patients at diagnosis (*n*=5) or at disease progression (*n*=3). Results showed that in asymptomatic WM at diagnosis, *MYD88^L265P^* was the predominant clonal alteration; other events, if present, were secondary and subclonal to *MYD88^L265P^*. In symptomatic WM, clonal diversity was more evident, uncovering combinations of alterations that synergized to promote clonal expansion and dominance. At disease progression, a dominant clone was observed, sometimes accompanied by other less complex minor clones, which could be consistent with a clonal selection process. Clonal diversity was also reduced, probably due to the effect of treatment. Finally, we combined protein expression with mutational analysis to map somatic genotype with the immunophenotype. Our findings provide a comprehensive view of the clonality of tumor populations in WM and how clonal complexity can evolve and impact disease progression.

## Teaser

**Summary:** Our study shows, for the first time, the clonal architecture of Waldenström's macroglobulinemia at the single-cell level, providing insight into how the oncogenic process can occur.

## INTRODUCTION

Waldenström's macroglobulinemia (WM) is a distinct, indolent, B-cell lymphoproliferative disorder characterized by bone marrow infiltration by lymphoplasmacytic lymphoma and the presence of an immunoglobulin M (IgM) monoclonal component (Dogliotti et al., 2023; Owen et al., 2003). The cellular composition of this IgM lymphoma is variable, including malignant small lymphocytes, plasmacytoid lymphocytes and plasma cells in variable percentages (Stone and Pascual, 2010). At the clinical level, the disease is consistently heterogeneous, with a behavior ranging from indolent forms, such as IgM monoclonal gammopathy of undetermined significance (IgM-MGUS) and asymptomatic WM, to highly symptomatic disease (symptomatic WM), with evolution being highly variable as well (Oza and Rajkumar, 2015).

Over the past decade, much progress has been made in the molecular understanding of WM through next-generation sequencing large-scale bulk analyses. Genomic characterization of WM tumor cells has identified recurrent somatic mutations in *MYD88* (>95% patients) and *CXCR4* (>30% patients) genes, and deletions involving chromosome 6q (del6q; ∼50% patients), among other alterations (Hunter et al., 2014; Schop et al., 2002; Treon et al., 2012). *MYD88^L265P^* mutation is considered to be the tumor-initiating event that provides an advantage for B-cell clonal selection and predisposes the malignant clone to further genetic alterations, leading to full-blown lymphoma development (Alcoceba et al., 2022; Argyropoulos et al., 2016; Sewastianik et al., 2019). However, most alterations are present in both symptomatic and asymptomatic WM, so the global genomic profile cannot explain the differences in the clinical behavior and evolution of the disease (Jiménez et al., 2018; Varettoni et al., 2017). The cell of origin of WM, the order of the events, their distribution in individual tumor cells and clones, and how these interact may be of great relevance to the course of the oncogenic process. However, by bulk sequencing, it is not possible to obtain that information because cell identities are not preserved. Innovative single-cell sequencing technologies allow the dissection of the tumor genetic heterogeneity and accurately measure clonal complexity, deciphering the patterns of somatic mutations across clonal populations (García-Sanz and Jiménez, 2021). As tumors are constantly evolving, they often contain mutations that are relatively rare when they first emerge. Detecting these mutations and the clones that carry them may be of clinical importance for minimal residual disease, therapeutic resistance, or disease progression and transformation (Demaree et al., 2021; Guess et al., 2022; Meyers et al., 2022; Nadeu et al., 2022; Robinson et al., 2022; Wang et al., 2022).

To date, there are few studies of WM at the single-cell level (Cholujova et al., 2023; Kaushal et al., 2021; Mondello et al., 2023; Rodriguez et al., 2022; Sun et al., 2022). Only one of these studies is based on DNA sequencing, and although it allowed the identification of the presence of *MYD88^L265P^* in B-cell precursors, it did not provide data on the order of mutation acquisition, co-mutation patterns, or how the mutational landscape fluctuates over the course of the disease (Rodriguez et al., 2022). Here, we performed an integrated single-cell DNA-sequencing and immunophenotyping study to establish the sequence of genetic events, the target populations in which they arise, and the co-dependency/exclusion of alterations. We analyzed the cell populations at baseline and following therapy to better understand the tumor architecture and evolutionary trajectories underlying the oncogenic process. The correlation with the immunophenotype provided information about the different cell populations present and whether they were part of the neoplastic clone.

## RESULTS

A total of 42,352 cells were used for single-cell analysis (median 3839 cells/patient; range, 1031-11307 cells). Patients' medical history and immunophenotype of the samples used are provided in Table 1.

**Table 1.**
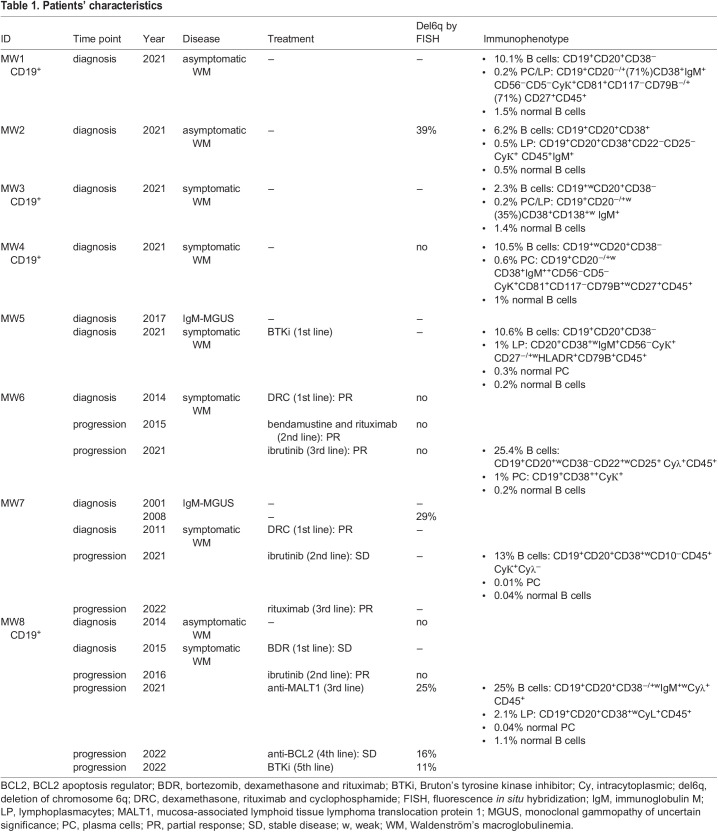
Patients' characteristics

### Tumor architecture and order of events

**Fig. 1.**
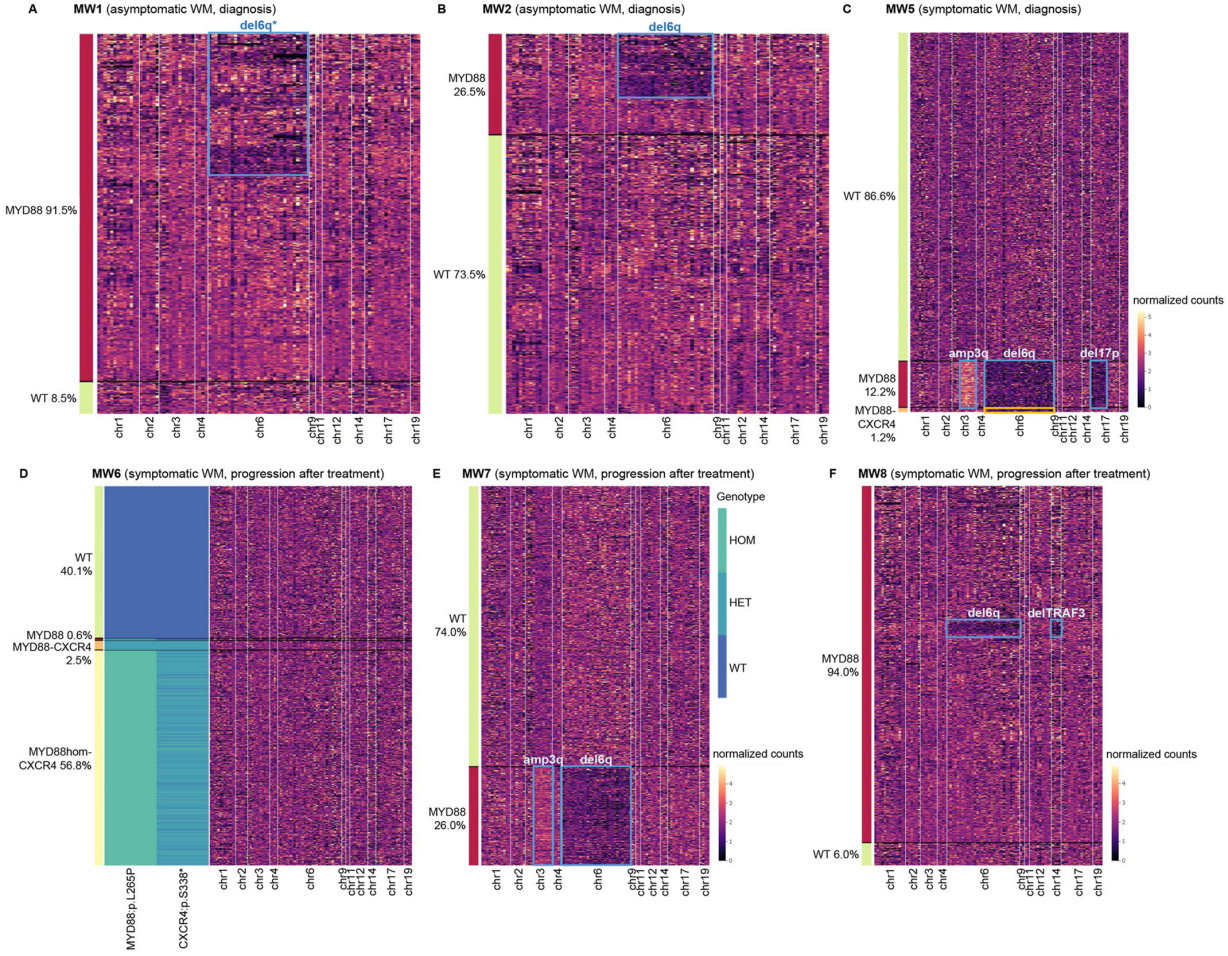
**Clonal architecture of different disease stages of Waldenström's macroglobulinemia (WM) at the single-cell level.** (A) Presence of wild-type (WT; green) versus heterozygous *MYD88^L265P^* (red) and deletion of 6q (outlined in blue) in an asymptomatic WM patient at diagnosis (MW1). (B) Presence of WT (green) versus heterozygous *MYD88^L265P^* (red) and deletion of 6q (outlined in blue) in an asymptomatic WM patient at diagnosis (MW2). (C) Distribution of somatic variants (*MYD88^L265P^* and CXCR4^S344*^) and copy number alterations (deletion of 6q, deletion of 17p and amplification of 3q) in a patient with symptomatic WM at diagnosis (MW5). (D) Distribution of somatic variants (*MYD88^L265P^*, heterozygous and homozygous, and CXCR4^S338*^) in one symptomatic WM patient at disease progression (MW6). (E) Distribution of somatic variants (heterozygous *MYD88^L265P^*) and copy number alterations (deletion of 6q and amplification of 3q) in one symptomatic WM patient at disease progression (MW7). (F) Presence of *MYD88^L265P^*, deletion of 6q and deletion of *TRAF3* in one patient with symptomatic WM at the time of disease progression (MW8). In all panels, rows represent the individual cells and columns represent the regions covered by the panel amplicons. Color scale indicates the number of normalized reads for copy number alterations.

First, analysis showed that *MYD88^L265P^* was the most clonal alteration at diagnosis. MW1 and MW2 were both diagnostic samples of asymptomatic WM. *MYD88* mutation defined the main tumor clone in these patients (it was present in 91.5% and 26.5% of cells, respectively). Part of the clone (∼25% and ∼16% of cells, respectively) had acquired del6q, confirming that it was a secondary alteration that appears after *MYD88* mutation (Fig. 1A,B). In MW3 and MW4 (both symptomatic WM at diagnosis), *MYD88^L265P^* was also clonal (80.4% and 89.7% of cells, respectively), but no further alterations were detected. MW5 was diagnosed as IgM-MGUS (in 2017) and, 4 years later, progressed to symptomatic WM. We analyzed the sample of progression to symptomatic WM. At that time, two subclones could be differentiated: *MYD88*-del6q-del17p-amp3q (12.2%, outlined in blue in Fig. 1C), and *MYD88*-del6q-*CXCR4* (1.2%, outlined in yellow in Fig. 1C). Both had in common *MYD88^L265P^* and del6q, but then one of the subclones acquired a *CXCR4* mutation, whereas the other one acquired del17p and amp3q. A branching model of disease evolution can be inferred based on this clonal distribution (Fig. 1C).

In samples taken at the time of disease progression, the scenario changed. The secondary alterations were (mostly) present in the same cells as *MYD88^L265P^*. MW6 was a symptomatic WM, and we analyzed the sample of progression after the second line of treatment. The main tumor clone of this patient (56.8% of cells) presented a *CXCR4* mutation and was homozygous for *MYD88^L265P^*, possibly due to an acquired uniparental disomy (aUPD) of chromosome 3 (chr3). Interestingly, there were two additional small subclones without the loss of the chr3 copy: one with the *MYD88* mutation only (0.6%), and the second one with both *MYD88* and *CXCR4* mutations (2.5%) (Fig. 1D). These two small clones could represent the initial clones, which shed light on how the oncogenic process might have occurred. MW7 was first diagnosed as IgM-MGUS and, 10 years later, progressed to symptomatic WM. The sample analyzed was the progression after the first line of treatment. The alterations this patient had (del6q and amp3q) co-occurred in the same cells as the *MYD88* mutation, and, in this case, there were no remaining cells representing any potential ancestor (Fig. 1E). Finally, MW8 was a patient diagnosed as asymptomatic WM, who progressed to symptomatic WM. The sample included corresponded to the time of progression after ibrutinib therapy (second line). This sample had a del6q that, according to fluorescence *in situ* hybridization (FISH) results, was not present in the samples at diagnosis or when the patient progressed to symptomatic WM. The subclone with this alteration (∼6.6% of the CD19^+^ cells), along with del*TRAF3*, may therefore be considered emergent at the time of ibrutinib progression (Fig. 1F). Predicted evolution of the events based on these observations is presented in Fig. S1.

### Co-occurrence and exclusion of alterations

Next, we investigated the co-dependency and exclusion of alterations at single-cell resolution. As mentioned, we detected the *MYD88^L265P^* variant in all patients (8/8), defining the main clone and supporting its role as the tumor-initiating event. Secondary oncogenic events, such as del6q (present in 5/8 patients), *CXCR4* mutations (2/8 patients), amp3q (2/8 patients), del*TRAF3* (1/8 patients) and del17p (1/8 patients), accompanied *MYD88* mutation. *CXCR4* mutations were subclonal to *MYD88* at diagnosis (MW5, Fig. 1C), but not at disease progression (MW6, Fig. 1D), as happened with amp3q (MW5, Fig. 1C; MW7, Fig. 1E). Del6q was subclonal to *MYD88* at diagnosis in asymptomatic WM (MW1 and MW2, Fig. 1A,B) but not in symptomatic WM (MW5, Fig. 1C). Finally, the aUPD of chr3 co-occurred with *CXCR4* mutation (MW6, Fig. 1D).

We also observed that alterations with a common role could concur in the same cells. Thus, deletions of two negative regulators of the NF-κB signaling pathway, *TRAF3* and *TNFAIP3* (i.e. del6q), co-occurred in the same subclone in one patient (MW8, Fig. 1F). Del6q (*TNFAIP3*) also co-existed with amp3q, which includes *TBL1XR1*, a gene involved in the activation of NF-κB, in two patients (MW5, Fig. 1C; MW7, Fig. 1E). By contrast, del6q always showed mutual exclusivity with *CXCR4* mutations, except in a minority subclone of one patient, suggesting that these are two different pathways that can promote disease progression. In the majority clone of this patient, del6q co-occurred with del17p and amp3q (MW5, Fig. 1C). However, all findings are based on very few numbers and therefore must be interpreted with caution.

### *MYD88^L265P^* in normal B cells

*MYD88* mutation has recently been found in immunophenotypically normal B cells of WM patients (Kaushal et al., 2021; Rodriguez et al., 2022). To confirm these findings, we compared the percentage of clonal B cells by flow cytometry (FCM), defined by the monoclonal light chain restriction, with the percentage of cells having *MYD88* mutation. For results to be comparable, we calculated the percentage of tumor cells out of the total mononuclear cells (in patients MW2, MW5, MW6 and MW7), and out of the total CD19^+^ cells in samples with CD19^−^ cell depletion (i.e. MW1, MW3, MW4 and MW8). Overall, numbers were similar, but patients MW2, MW3 and MW6 presented a higher percentage of *MYD88*-mutated cells (26.5%, 80.4% and 59.9%, respectively) than clonal cells (B lymphocytes and plasma cells) by FCM (20.4%, 63% and 45.2%, respectively), which concurs with the existence of *MYD88*-mutated non-clonal cells.

### Single-cell protein-sequencing analysis

Protein analysis was based on the expression of CD34, CD19, CD20 (MS4A1) and CD38 antigens. Protein libraries of patients MW2 and MW4 failed (so no expression data were available for these cases), and anti-CD19 antibody did not work in MW7. According to FCM data, immature cells (named B-cell precursors) were defined as CD34^+^CD19^+^CD20^−^CD38^+^, B lymphocytes as CD34^−^CD19^+^CD20^+^CD38^−^, and plasma cells/plasmacytoid lymphocytes as CD34^−^CD19^+^CD20^+^CD38^+^. Cells with a phenotype not consistent with any of the above were removed from the final plots. Clonality of B cells could not be assessed based on these markers.

**Fig. 2.**
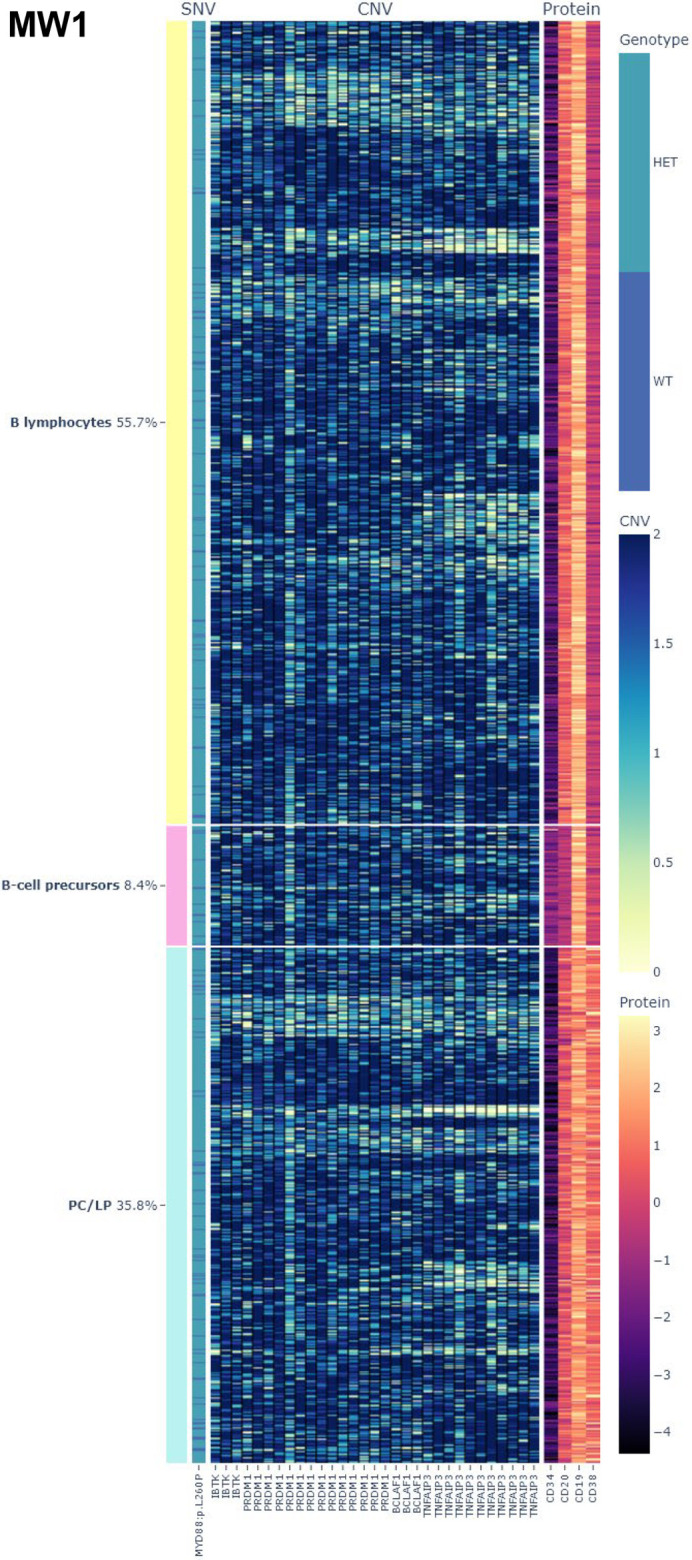
**Copy number alterations in selected regulatory regions of 6q in one patient with asymptomatic WM.** Rows represent the individual cells and columns represent the amplicons of 6q region. Yellow-blue color scale indicates the copy number alterations. The number of deleted amplicons/genes differed between cells, with *TNFAIP3* the most frequently deleted gene. CNV, copy-number variation; HET, heterozygous; LP, lymphoplasmacytes; PC, plasma cells; SNV, single-nucleotide variant; WT, wild type.

Despite these difficulties, analysis showed that alterations were present in all cell populations, and, likewise, all populations had wild-type cells (at least for the alterations evaluated with this panel) (Figs S2 and S4). In addition, slight phenotypic differences could be observed in mutated versus wild-type cells within each population (Fig. S2) and in all cells in patients in whom CD19 detection was not performed (Fig. S3): MW5 (CD19 and CD38 expression), MW6 (CD19, CD38 and CD34 expression) and MW7 (CD20 and CD38 expression). Thus, mutated cells expressed CD19 or CD20 more strongly, whereas CD34 and CD38 expression was weaker than in unmutated cells (Fig. S3). In samples with depletion of CD19^−^ cells (MW1, MW3 and MW8), these differences could not be so well appreciated because most cells were *MYD88* mutated (Figs S4 and S5). In MW1, when we clustered the cells based on protein expression, we could observe that the 6q region studied (genes *IBTK*, *PRDM1*, *BCLAF1* and *TNFAIP3*) was not completely and equally deleted in all cells, appearing that *TNFAIP3* was the first gene to be deleted and the most common in the immature cells (Fig. 2).

## DISCUSSION

The genomic landscape of WM has been well described, and has allowed the characterization of disease mechanisms and the identification of biomarkers and therapeutic targets (Braggio et al., 2009; Hunter et al., 2014; Jiménez et al., 2018; Poulain et al., 2013; Schop et al., 2002; Treon et al., 2012; Varettoni et al., 2017). By bulk sequencing, it is possible to infer patterns of clonality, sub-clonality and clonal evolution using variant allele frequency (VAF) distribution. However, single-cell techniques can provide more detailed and direct approaches to study intratumor heterogeneity and clonal architecture. Here, we have performed a single-cell DNA- and protein-sequencing study in WM with the Tapestri platform. Single-cell analysis assigns alterations to different clones, allowing the reconstruction of tumor evolutionary histories and identification of disease-initiating events, as well as cooperative mutations that give cells a fitness advantage (Navin et al., 2011).

Genetic evidence supports a stepwise accumulation of genomic alterations (mutations, copy-number abnormalities, loss of heterozygosity) during WM development, suggesting that they have a role in the multistep oncogenic process that drives this transition (Jiménez et al., 2018; Paiva et al., 2015; Poulain et al., 2013; Varettoni et al., 2017). Thus, we found that clonal complexity increased as disease evolved, but that could also be reduced owing to the effect of treatment. The presence of more mature subclonal populations with a higher number of genetic alterations (*MYD88*, *CXCR4*, del6q, del17p, amp3q) was associated with more advanced and symptomatic disease that required treatment. Considering the distribution of the alterations within the different subclones and their respective clonality, we proved that *MYD88^L265P^* was the common driver event and that only clones containing *MYD88* mutation gave rise to more aggressive populations by acquiring new alterations. Depending on the clone in which they arise, these alterations may give certain cells a fitness advantage, resulting in the intraclonal heterogeneity seen in WM and the different models of tumor evolution (Bolli et al., 2014; Greaves and Maley, 2012). Patient MW5 could represent an example of branched evolution, as two separated subclones (one with del17p and amp3q, and the other with *CXCR4* mutation) derived from a common ancestor that harbored *MYD88^L265P^* and del6q. The expansion of both clones is restrained by a mutual competition known as clonal interference (Anderson et al., 2011).

Previous studies suggest that there might be at least two distinct oncogenic pathways that promote progression to symptomatic disease in WM: mutated *CXCR4* and del6q (Cao et al., 2015; Guerrera et al., 2018; Hunter et al., 2016; Roccaro et al., 2014). Loss of chromosome 6q is found in 40-50% of patients with WM and appears exclusive of *CXCR4* in treatment-naïve patients, suggesting shared roles for the two genomic events (Guerrera et al., 2018; Schop et al., 2002). We found del6q in 5/8 patients, showing mutual exclusivity with *CXCR4* mutations in all but one patient (MW5), and, even in this case, the subclone in which both alterations co-occurred was minority. The predominant subclone of this patient had del6q, amp3q and del17p, suggesting that these are cooperative alterations that provide cells a proliferative advantage. We also found other alterations that induce the same mechanism, such as del6q and del*TRAF3*, or del6q and amp3q, all of them leading to activation of the NF-κB signaling pathway, co-existing in the same patients and even in the same subclones (Braggio et al., 2009; Jung et al., 2017).

The changes in the tumor architecture observed at disease progression could be attributed to a cancer-clone evolutionary selection for more robust or malignant phenotypes (Greaves and Maley, 2012). In patient MW6, the few remaining cells having fewer alterations (*MYD88^L265P^* alone or *MYD88^L265P^* plus *CXCR4* mutation) than the main clone could represent the initial clones, illustrating how the oncogenic process may have occurred: *MYD88^L265P^* was the initial oncogenic event, then *CXCR4* mutation was acquired and, finally, the aUPD of chr3, both being present in the same subclone. The aUPD may have potentially contributed to clonal evolution by rendering tumor cells homozygous for a pre-existing oncogenic mutation (*MYD88^L265P^*) (Treon et al., 2016). In MW7, the scenario was similar, but because no remnants of the potential initial clones could be observed, the temporal ordering of genomic events could not be inferred. Both functionally relevant mutational events and therapy can drive clonal selection, but to gain more detailed insight into clonal trajectories in individual patients, this issue needs to be best addressed by serial sampling (Bolli et al., 2014; Greaves and Maley, 2012). Our last case (MW8) may exemplify the emergence of new clones resistant to therapy. Del6q has been associated with disease progression (García-Sanz et al., 2021) and ibrutinib resistance in WM (Jimenez et al., 2020). Because this alteration was not present in the previous moments (according to FISH), it could be hypothesized that the subclone with del6q and del*TRAF3* is responsible for the treatment resistance acquisition, especially considering that the patient did not have mutations in *BTK* (Xu et al., 2017). However, considering that the sensitivity of FISH does not reach the single-cell level, we also cannot discard the possibility that new selective pressures (i.e. ibrutinib therapy) had allowed pre-existing cancer cells that survived treatment to emerge. Alterations in *CXCR4* have also been reported as associated with drug resistance, including resistance to ibrutinib (Cao et al., 2015; Roccaro et al., 2014; Treon et al., 2015). In our series, only two patients harbored these alterations but did not show treatment resistance.

Single-cell techniques allow not only the establishment of the order of the events, but also the sequence of acquisition of structural variants. In the asymptomatic WM (MW1), among the genes we evaluated, *TNFAIP3* seemed to be the most frequently deleted gene, which suggests that del6q begins to happen around this area. In symptomatic WM, the entire 6q region evaluated was equally deleted.

In contrast to other works (Rodriguez et al., 2022), we did not find wild-type *MYD88* in tumor cells carrying other genetic lesions, at least for the alterations we studied. However, based on the comparison of the tumor infiltration by FCM and the percentage of cells carrying *MYD88* mutation by single-cell analysis, we were able to confirm that this alteration can be present in phenotypically normal B cells of WM patients (Rodriguez et al., 2022). Emerging evidence has suggested that *MYD88^L265P^* would arise during hematopoietic development, although not always at the same cellular stage, and that parallel clonal expansions occur before subclones begin to dominate in early cancer development but are rare after cell transformation (Anderson et al., 2011; Rodriguez et al., 2022; Siegmund et al., 2009). Most somatic mutations present in progenitor cells are undetectable in mature B lymphocytes, suggesting continuous clonal selection until oncogenic alterations appear and cause the transformation. We observed slight differences in the immunophenotype of *MYD88*-mutated compared to wild-type cells. Therefore, one might think that the acquisition of *MYD88* mutation would be accompanied by changes in the immunophenotype as the B-cell clone progressively grows and evolves. The immune microenvironment has been shown to play a critical role in this transition (Kaushal et al., 2021).

Single-cell data may have biological and therapeutic relevance in the future. Intratumor heterogeneity has been shown to be prognostic in certain cancer types and can also explain the partial efficacy of targeted therapies (Andor et al., 2016; Landau et al., 2013; Rocco, 2015; Yates et al., 2015) or why it is impossible to achieve complete responses with BTK inhibitors as monotherapy in WM (Buske et al., 2022; Tam et al., 2020). Current treatment approaches typically consider the disease to be static and homogeneous. Understanding the heterogeneity within tumors and their ability to evolve in response to therapy could allow the design of interventions to disrupt clonal evolution and/or target WM as a multi-clonal disease. Also, the identification of subclones potentially associated with treatment resistance may allow the use of preventive therapeutic strategies.

We are aware of the limitations of our study, especially the number of patients, not having sequential samples, the gene panel size and the use of a new methodology. Nevertheless, our results are supported by FISH and FCM studies.

In summary, we have precisely characterized the clonal architecture of WM at the single-cell level for the first time. Our work including the different stages of WM (asymptomatic, symptomatic and symptomatic post-therapy) provides information about how disease initiates and progresses, the timing of the alterations (early versus late), the cooperative mutations and the patterns of evolution.

## MATERIALS AND METHODS

### Patients and samples

Eight WM patients – five at diagnosis and three at the time of disease progression – were included in the study. Cases were diagnosed using standard World Health Organization classification criteria (2016 update; Swerdlow et al., 2016), and diagnoses were fully concordant with the new updates (Alaggio et al., 2022; Campo et al., 2022). Samples were selected based on the tumor infiltration detected by FCM during the standard diagnostic process. Mononuclear cells were isolated from bone marrow by Ficoll-Paque density-gradient centrifugation, and CD19^−^ cells were removed with an EasySep™ Human B Cell Enrichment Kit (STEMCELL Technologies) when sufficient cells were available (patients MW1, MW3, MW4 and MW8). Cells were preserved in fetal bovine serum with 10% dimethyl sulfoxide at −80°C until use in single-cell studies. Previous and/or subsequent samples from these patients were evaluated by FISH, FCM and molecular studies to help understand the evolutionary history of the tumors. The study was approved by the ethics committee following the ethical recommendations and guidelines of the Declaration of Helsinki. Written informed consent was obtained from all eight patients.

### Single-cell DNA and protein sequencing

We designed a Tapestri™ Single-Cell DNA Custom Panel of 112 amplicons (Mission Bio, San Francisco, CA, USA) covering the hotspot regions of 20 genes [*MYD88*, *CXCR4*, *ARID1A*, *KMT2D*, *TP53*, *CD79A*, *CD79B*, *NOTCH2*, *TRAF2*, *TRAF3*, *MYBBP1A*, *HIST1H1E* (*H1-4*), *KLF2*, *TBL1XR1*, *PTPN13*, *RAG2*, *IBTK*, *PRDM1*, *BCLAF1* and *TNFAIP3*, for the assessment of 6q deletion], and a panel of oligonucleotide-conjugated antibodies targeting the following surface proteins: CD19, CD20, CD34, CD38 and CD138 (SDC1). CD34 oligonucleotide-conjugated antibody was used in a 1:2 dilution, CD38 oligonucleotide-conjugated antibody was used in a 1:5 dilution, and the remaining antibodies were used without dilution. Owing to its overexpression compared to the other proteins, CD138 was removed from the final analysis.

Simultaneous profiling of DNA mutation and cell-surface immunophenotype was performed according to the manufacturer's protocol. Cell suspensions had to contain 6000-10,000 cells/µl at ≥80% viability to be processed. Briefly, cryopreserved bone marrow mononuclear cells were thawed, quantified and stained with the pool of five oligonucleotide-conjugated antibodies. The stained cells were washed and loaded into the Tapestri instrument for single-cell encapsulation, lysis and barcoding. Targeted amplification using multiplexed PCR occurred within the droplets. DNA and protein PCR products were then isolated from the individual droplets, purified with AMPure XP beads (Beckman Coulter) and streptavidin beads (Thermo Fisher Scientific), and used for library generation. DNA- and antibody-tagged final libraries were quantified using a Qubit 4 fluorometer (Thermo Fisher Scientific) and pooled for sequencing on a NextSeq 1000/2000 sequencer (Illumina, San Diego, CA, USA) using 2×150 bp cycles.

### Data analysis

Raw FASTQ files were processed with the Tapestri pipeline v2 (Mission Bio), which includes adapter trimming using Cutadapt, alignment to the reference human genome GRCh37/hg19 using Burrows-Wheeler Aligner, cellular barcode demultiplexing, and cell-based genotype calling using GATK/HaplotypeCaller. The output .loom files were analyzed using Tapestri Insights (v2.2), filtering out low-quality genotypes and cells (i.e. genotype quality <30, reads/cell/target <10, mutant VAF <20%, variants mutated in <1% of the cells, and cells with <50% of genotypes present). The python-based Mosaic analysis package (https://github.com/MissionBio/mosaic) was used for more advanced multiomics analysis and data visualization of the .h5 files. Only cells with complete genotype information of the variants selected for downstream analysis were included.

### FCM immunophenotyping

Bone marrow samples were processed following the general recommendations of the EuroFlow group (Kalina et al., 2012) and stained with at least an eight-color panel including monoclonal antibodies against the following antigens combined in several tubes: surface immunoglobulin-M (SIgM), CD5, CD19, CD20, CD22, CD23 (FCER2), CD25 (IL2RA), CD27, CD38, CD45 (PTPRC), CD56 (NCAM1), CD79B, CD81, CD117 (KIT), CD138, and intracytoplasmic IgM (CyIgM), kappa (CyК) and lambda (Cyλ). A minimum of 1 million cells were acquired per tube in a FACSCalibur flow cytometer (Becton Dickinson Biosciences, San Jose, CA, USA) using BD FACSDiva™ software v6.1 (Becton Dickinson Biosciences). Data were analyzed using Infinicyt™ software v2.0 (Cytognos, Salamanca, Spain). Light chain-restricted clonal lymphocytes (CD19^+^) and plasma cells (CD38^+^ or strong CD138^+^) were quantified (Paiva et al., 2014; Puig et al., 2017).

### FISH studies

Simple interphase FISH was performed on cell nuclei of CD19^+^ cells from bone marrow samples using our previously published techniques (Ocio et al., 2005). Deletions of 6q and 17p, and translocations of 14q32 were evaluated. At least 100 cells were analyzed in all samples, applying Vysis scoring criteria (Abbott Laboratories, Abbott Park, IL, USA). The cutoff point for the identification of an alteration was set at ≥10% cells with an abnormal signal.

## Supplementary Material

10.1242/dmm.050227_sup1 Supplementary information
