# Supplementary material for: Clonal architecture and evolutionary history of Waldenström's macroglobulinemia at the single-cell level
Source: Dis Model Mech. 2023 Aug 23;16(8):dmm050227. doi: 10.1242/dmm.050227 (PMC10461465; doi:10.1242/dmm.050227)
Supplement: Supplementary information [file dmm-16-050227-s1.pdf]

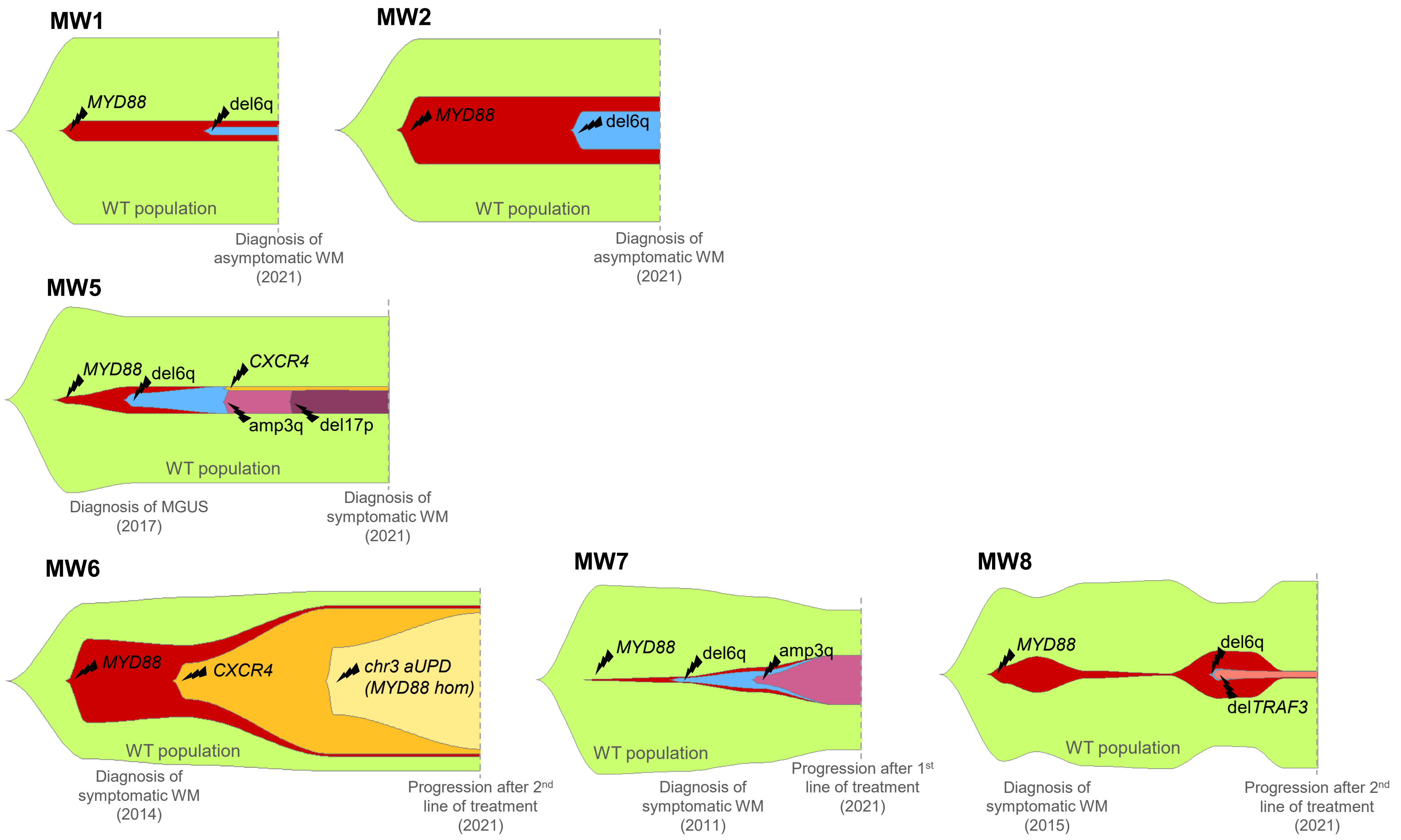

Amp3q, amplification of chromosome 3q; chr3 aUPD, acquired uniparental disomy of chr3; del17p, deletion of chromosome 17p; del6q, deletion of chromosome 6q; del*TRAF3*, deletion of *TRAF3* gene (chromosome 14); hom, homozygous for *MYD88*^{L265P}; WM, Waldenström's macroglobulinemia; WT, wild-type

Fig. S1. Fishplots representing the hypothesized evolution of the clones in eight Waldenström's macroglobulinemia patients. The proposed order of the events was inferred based on single-cell data, FISH results, and the patients' clinical history.

Disease Models & Mechanisms • Supplementary information

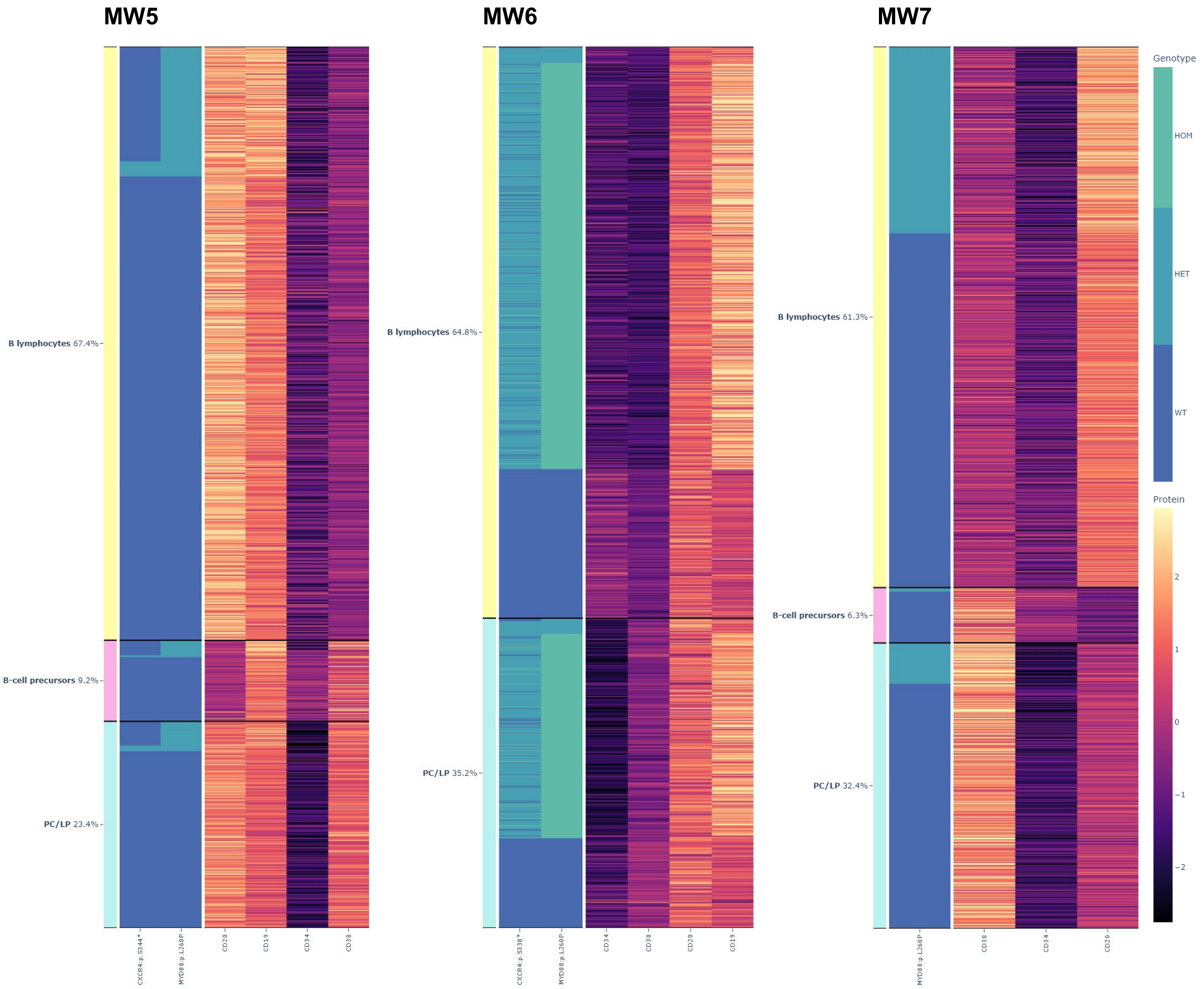

Het, heterozygous; Hom, homozygous; PC/LP, plasma cells/plasmacytoid lymphocytes; WT, wild-type

Fig. S2. Combined representation of genotype and phenotype of Waldenström's macroglobulinemia single cells clustered by phenotype. Cells were clustered based on the expression of CD19, CD20, CD34 and CD38 antigens and designated as the different cell populations (B lymphocytes, B-cell precursors, and plasma cells). Status of the somatic variants (*MYD88* and *CXCR4*) within each population is represented in green and blues. Rows represent the individual cells and columns represent the somatic mutations and the antigen expression. Color scale indicates the intensity of antigen expression.

Disease Models & Mechanisms • Supplementary information

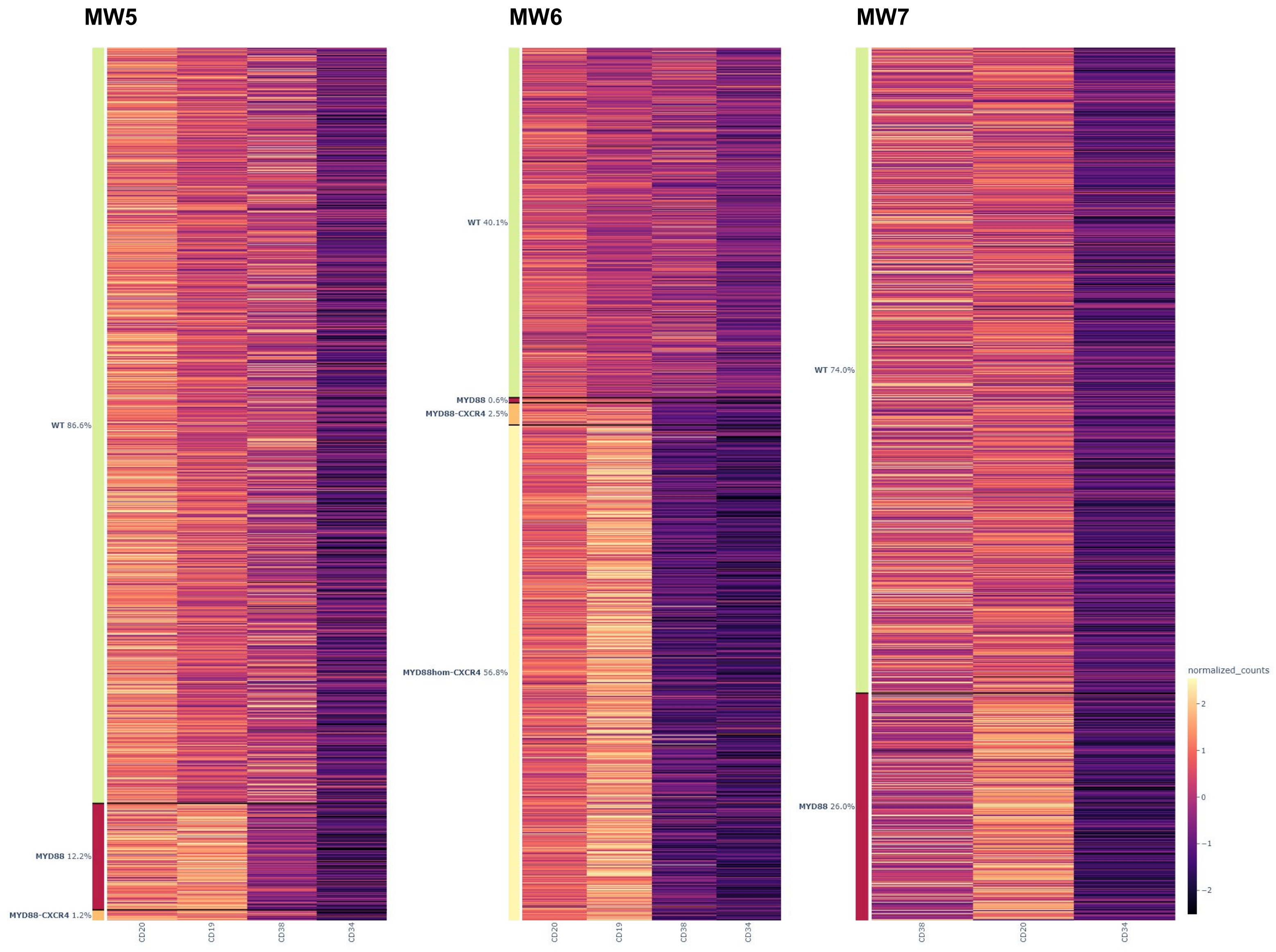

Hom, homozygous; WT, wild-type

Fig. S3. Combined representation of genotype and phenotype of Waldenström's macroglobulinemia single cells clustered by genotype. Expression of CD19, CD20, CD34, and CD38 is different in (*MYD88* and *CXCR4*) mutated cells compared to wild-type cells. Rows represent the individual cells and columns represent the antigen expression. Color scale indicates the intensity of antigen expression.

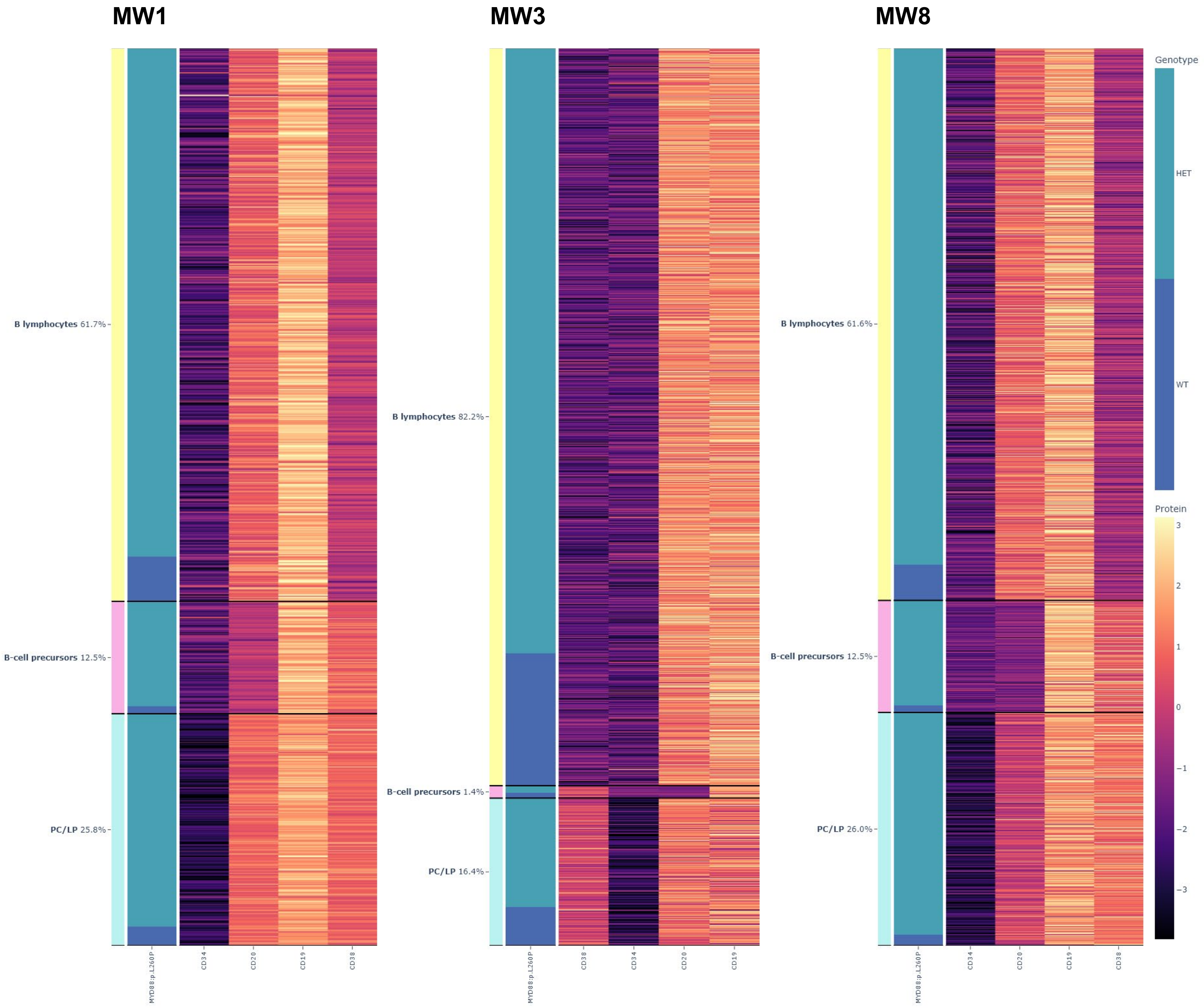

Het, heterozygous; PC/LP, plasma cells/plasmacytoid lymphocytes; WT, wild-type

Fig. S4. Combined representation of genotype and phenotype of Waldenström's macroglobulinemia single-cells clustered by phenotype. Cells were clustered based on the expression of CD19, CD20, CD34 and CD38 antigens and designated as the different cell populations. Status of the somatic variant (*MYD88*^{L265P}) within each population is represented in green and blue. Rows represent the individual cells and columns represent the somatic mutations and the antigen expression. Color scale indicates the intensity of antigen expression. As these were CD19 negative depleted samples, most cells were *MYD88*-mutated, and no differences in the antigen expression could be observed based on the genotype.

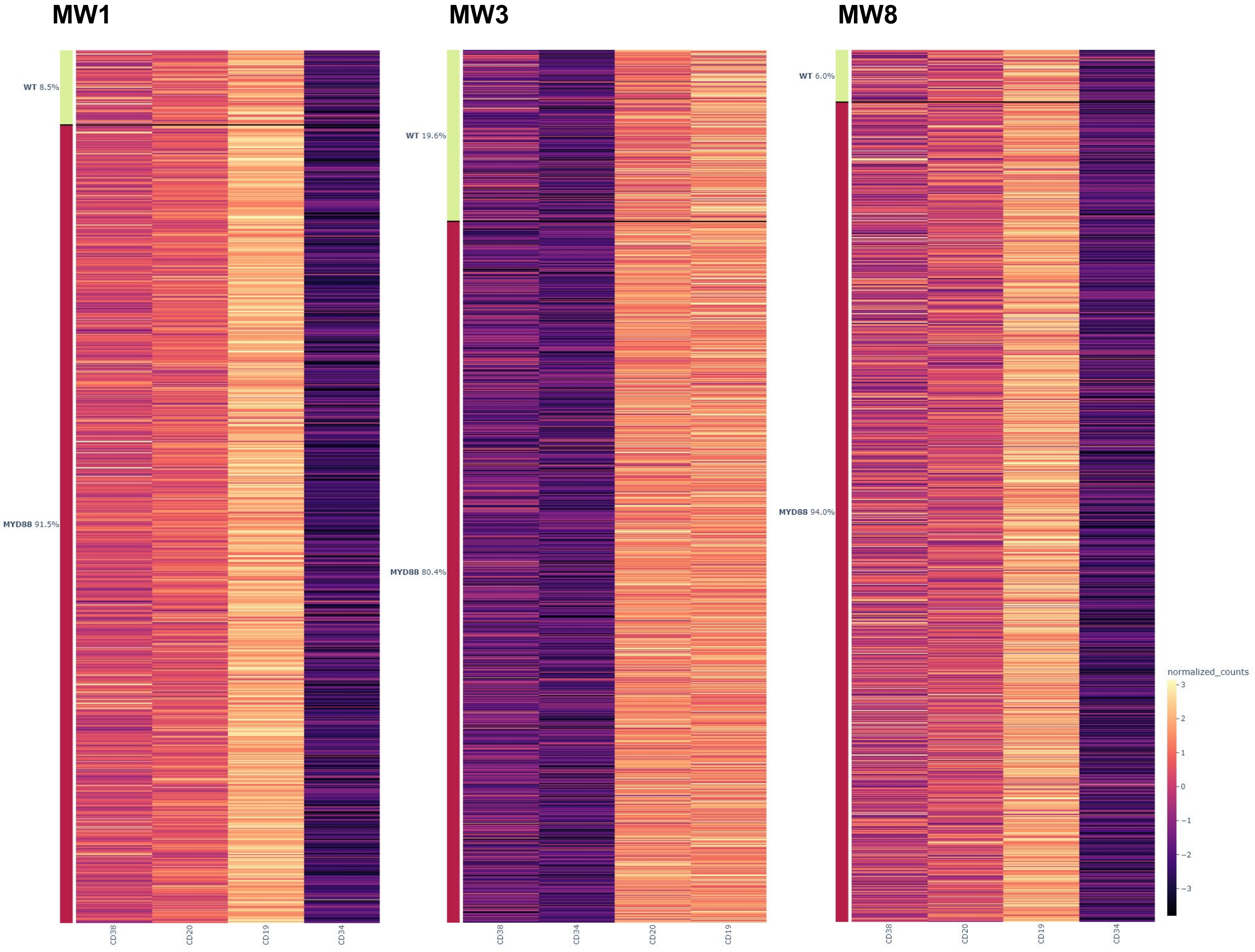

WT, wild-type

Fig. S5. Combined representation of genotype and phenotype of Waldenström's macroglobulinemia single cells clustered by genotype. As these were CD19 negative depleted samples, most cells were *MYD88*-mutated, and no differences in the antigen expression could be observed in mutated cells compared to wild-type cells. Rows represent the individual cells and columns represent the antigen expression. Color scale indicates the intensity of antigen expression.
